# Intersectional inequalities in social-emotional problems among three-year-old children in Sweden: a population-based study

**DOI:** 10.1186/s12889-026-27220-8

**Published:** 2026-04-09

**Authors:** Ana Rosenberg, Anneli Ivarsson, Marie Lindkvist, Sven-Arne Silfverdal, Masoud Vaezghasemi

**Affiliations:** 1 Department of Epidemiology and Global Health, Umeå University, Umeå, Sweden; 2 Department of Clinical Science, Pediatrics, Umeå University, Umeå, Sweden

**Keywords:** Social-emotional problems, Mental health, Preschool children, Social inequalities, Intersectionality, Discriminatory accuracy, Proportionate universalism, Sweden

## Abstract

**Background:**

Social-emotional development is an important part of early childhood development and appears to have a socioeconomic gradient. Going beyond conventional approaches, this study aimed to examine intersectional inequalities in social-emotional problems in three-year-old children in relation to their parents’ income, education and place of birth and to consider the implications for public health.

**Methods:**

A cross-sectional design was used in this population-based study in Västerbotten County of Sweden with an effective sample of 8,823 children. Social-emotional problems were assessed using the parent/caregiver-report Ages and Stages Questionnaires: Social-Emotional (ASQ:SE) 36-month interval in Child Health Services over the years of 2014–2018 and linked to parents’ sociodemographic characteristics obtained from national population registers. An analysis of individual heterogeneity and discriminatory accuracy (AIHDA) approach was combined with additive binomial regression, estimating risk differences for social-emotional problems across 27 intersectional categories.

**Results:**

In the intersectional categories where multiple dimensions of social disadvantage overlapped, average risk differences generally increased. For instance, when comparing with the most advantaged category a risk difference as high as 25.4% (95% CI 13.7 to 37.0%) was found for the children whose parents’ income was in the lowest tercile, only one parent had a higher educational level and whose parents were both born outside of Sweden. Discriminatory accuracy was estimated as moderate for the three included regression models, although it improved slightly for the model including dimensions of social disadvantage. The addition of intersectional categories provided no further significant improvement.

**Conclusions:**

The intersectional approach used in this study improves our understanding of complex social inequalities in social-emotional problems in preschool children in northern Sweden. Consistent with the concept of proportionate universalism, the results of this study indicate that universal public health policies are needed when addressing this issue in addition to policies targeting disadvantaged groups. Research that considers individual heterogeneity and discriminatory accuracy has the potential to advance our knowledge of health inequities and increase the effectiveness of public health policy.

**Supplementary Information:**

The online version contains supplementary material available at 10.1186/s12889-026-27220-8.

## Background

Early childhood development, including social-emotional aspects, is a major contributing factor to lifetime opportunities, affecting such essential areas as health, schooling and societal participation [1]. Social-emotional development in early childhood involves the development of social-emotional competence, encompassing such constructs as emotional expressiveness and understanding, emotional and behavioural regulation, social problem-solving and relationship skills [2], while an atypical development of age-appropriate social-emotional competencies indicates social-emotional and behavioural problems [3]. Social-emotional and behavioural problems in young children are associated with mental health problems later in childhood [4–7], as well as having been negatively linked to academic achievement [8–11]. Early childhood social competence has also been associated with academic achievement, stable employment and mental health in young adults, in addition to having been found protective in relation to criminal activity [12].

The social determinants of health refer to the structures in society and conditions of life that cause systematic differences in health, creating a social gradient of health related to socioeconomic position [13]. In both a Swedish and an international context, socioeconomic status has been found to be related to social-emotional competence and problems in young children, including indicators such as family income or financial situation [14–18], and parental educational achievement [17–20]. Additionally, having parents with an immigrant background has been associated with an increased risk of social-emotional and behavioural problems [18, 20, 21]. Socioeconomic disadvantage has moreover been found to affect mental health the most during early childhood compared to later in life [22]. Social-emotional and behavioural problems in young children have also been associated with living with a single parent [17, 23, 24] as well as with an increased risk in boys compared to girls [14, 21, 23].

Research on inequalities in health often focuses on individual or even multiple social determinants, without taking the interactions of or connections between these into consideration [25]. Intersectionality first emerged as a term in an article by Kimberle Crenshaw in 1989 in relation to the interaction of race and gender and the discrimination of women of colour [26]. An intersectional approach to public health focuses on the most vulnerable and disadvantaged populations and involves seeing beyond individual factors such as gender or socioeconomic status and instead concentrating on the multiplicity and intersection of both individual-level and structural factors [27]. In addition to offering a framework for the study of health disparities, benefits of an intersectional approach may include encouraging more complex and multidimensional approaches to health inequalities as well as supporting more efficient health promotion through its focus on both disadvantaged groups and structural factors [27]. By taking heterogeneity within population groups into account, it may also avert research that lacks real-world implications [25].

In relation to intersectionality and the ways in which it may contribute to public health, the importance of taking the discriminatory ability of the intersectional approach into account has been argued [28]. Although average estimates of differences in risk of specific health outcomes in relation to certain risk factors are commonly used, they do not necessarily represent the most accurate way of classifying individual risk of the outcome [29]. From a public health perspective with regards to the improvement of population health, the relevance of a categorisation of exposure should be based on its ability to discriminate between those with the specified outcome and those without, rather than on an average association that may represent groups including large individual heterogeneity [30].

Thus, an intersectional approach has the potential to increase our understanding of complex inequalities and contribute to the development of more relevant public health policy. This study aimed to examine intersectional inequalities in social-emotional problems in three-year-old children related to their parents’ income, education, and place of birth in a Swedish context and additionally to consider the public health implications of an intersectional approach to this health issue.

## Methods

### Study design and context

This study is population-based with a cross-sectional design, conducted within the Salut Child Health Intervention Program. Data collection took place during the years of 2014–2018 and pooled to create one sample. The data consists of the Ages and Stages Questionnaire: Social-Emotional (ASQ:SE) [31], in addition to family sociodemographic characteristics secured by linking participants with national population registers from the government agency Statistics Sweden using personal identity numbers.

The Salut Child Health Intervention Program is a health promoting program in Västerbotten County in northern Sweden that was initiated in 2005. In addition to targeting other aspects of child health, it is integrated into existing maternal and child health care services. These health services are free in Sweden and include regular visits from pregnancy over birth until six years of age [32]. Prior to the three-year-old appointment in Västerbotten County, parents are given the Salut questionnaire including the ASQ:SE [31]. This is a screening instrument designed to identify young children with social-emotional problems by use of a standard cut-off point at which the intention is that children are referred for further assessment [33]. Additionally, the introduction of the ASQ:SE into Child Health Services in Västerbotten was motivated by an ambition to increase awareness of children’s social-emotional development in both parents and nurses. Areas of social-emotional competence that are central in the instrument include social interactions, affect, autonomy, adaptive behaviours, communication, compliance and self-regulation [33]. It has been found to suitably identify social-emotional problems in this age group in the local context, an evaluation that included examining internal consistency and unidimensionality [34]. Good accuracy has also been demonstrated in comparison with the Strengths and Difficulties questionnaire (SDQ) [35]. A Swedish translation of the first edition ASQ:SE 36-month interval was used, although the original English questionnaire was also available and used for parents who were more comfortable in English than Swedish. The translation had been done in accordance with recommendations [36].

### Study population

**Fig. 1:**
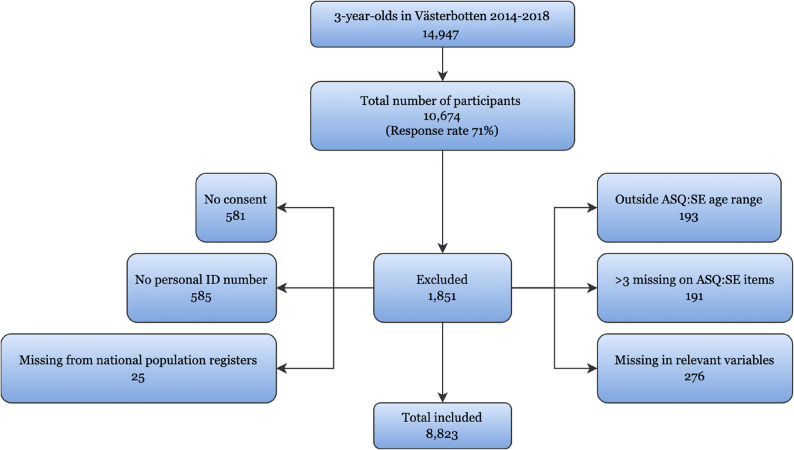
Flow chart of study participant exclusion

Among 14,947 children aged 3 years in Västerbotten County during the years 2014–2018 [37], the response rate to the Salut questionnaire was 71% which was equal to 10,674 children. Children were eligible for the study if they had attended the routine three-year-old child health care appointment during these years, were within the age range recommended for the ASQ:SE 36-month interval [33] and their primary caregivers had answered the Salut questionnaire. Following the exclusion process, which is visualized in Fig. 1, the effective sample consisted of 8,823 children.

### The outcome measure

The outcome social-emotional problems was defined as being above the cut-off score for the ASQ:SE indicating a risk for social-emotional problems [33]. The established US cut-off score for the ASQ:SE 36-month interval is 59 [33] and an outcome variable was created and dichotomized according to this definition. A cut-off of 50 has been found to be more appropriate for the local Swedish population in order to increase sensitivity [35] and all statistical analyses were also run for a second outcome based on this cut-off. See Additional File 1 for the results of these analyses.

The ASQ:SE 36-month interval consists of 31 scored items with three response alternatives relating to the frequency with which parents have registered a certain behaviour as well as if they are concerned by the behaviour. Point values of 0, 5 or 10 are assigned for the responses. An average score of available items was used to substitute up to three missing items as outlined in the ASQ:SE User’s Guide [33]. The questionnaire also includes three open-ended questions which were not used in this study.

### Explanatory variables

Parents’ income, education and place of birth were obtained from national population registers and examined as dimensions of social disadvantage and thereby social determinants of health. The average of parents’ disposable income per consumption unit (in Swedish SEK) for the three years prior to the child turning three years old [38], was adjusted for inflation using the Consumer Price Index to the common reporting year of 2017. This average of parents’ income was then categorised by tercile. In the context of this study, disposable income, including labour income and capital income in addition to other sources of income, is the capital that remains after paying taxes and social security fees. Parents’ education is a variable with three categories relating to the educational achievement of each parent: both more than high school, one more than high school and none more than high school. Finally, the variable of parents’ place of birth consists of three categories pertaining to where the parent was born: both born in Sweden, one born in Sweden, and none born in Sweden.

Data regarding the child’s sex, place of residence and custody arrangement was obtained from the Salut questionnaire at the three-year old appointment. Child’s sex was treated as a binary variable with the categories of boy or girl. Place of Residence is one of three parts of Västerbotten County where the child lived when the Salut questionnaire was answered, Södra Lappland, Skellefteå or Umeå. Custody arrangement is a binary variable, the child either lived with both parents or did not live with both parents.

### The intersectional cross-classified variable

An intersectional variable was constructed from the previously described variables concerning dimensions of social disadvantage: parents’ income, education and place of birth [39–41]. These were coded from 1 to 3 with 1 representing the most advantaged social position and 3 representing the most disadvantaged social position in each dimension. They were then cross-classified with each other creating 27 unique intersectional categories which allowed for the investigation of how the intersections of the different dimensions of social disadvantage were related to the defined outcome of social-emotional problems. The category of 111 therefor represents children with parents in the highest income tercile, who both had higher education and who were both born in Sweden. The category of 333 instead represents children whose parents were in the lowest income tercile, neither were educated past high school and who both were born outside of Sweden.

### Statistical analysis

The characteristics of the study population were descriptively examined through numbers and percentages, including the distribution of the intersectional categories. Additionally, the prevalence of the outcome was examined, in the whole study population as well as by characteristic. Complete case analysis was the chosen approach to handling missing data, as it constituted less than 2% for each explanatory variable and demonstrated a sufficiently random pattern.

The statistical analysis in this study was guided by an intersectional perspective to social inequalities in health, by using an analysis of individual heterogeneity and discriminatory accuracy (AIHDA) approach [39, 41]. AIHDA involves not only estimating average differences between groups but also examining the discriminatory accuracy of models, to assess the individual heterogeneities around average estimates [39, 41]. Risk differences for the outcome were estimated through a generalized linear model with a binomial family distribution using an identity link function [40, 42].

Three adjusted regression models were run separately for the outcome of social-emotional problems, based both on the ASQ:SE cut-off of 59 as well as 50. In the first model (Model 1) only the covariates child’s sex, custody arrangement and place of residence were included. In the second model (Model 2) the individual dimensions of social disadvantage parents’ income, parents’ education as well as parents’ place of birth were included in addition to the previously specified covariates. Finally, the third model (Model 3) consisted of the intersectional cross-classified variable as well as the covariates child’s sex, custody arrangement and place of residence. The collinearity of the included variables was examined, but the variance inflation factors did not indicate any such concerns. The significance of findings was determined using a 5% significance level.

Discriminatory accuracy was estimated through the area under the receiver operating characteristic curve (AUC) for the three previously described regression models [39–41]. An AUC = 0.5–0.6 was then interpreted as ‘absent/very small’, 0.6 < AUC ≤ 0.7 as ‘moderate’, 0.7 < AUC ≤ 0.8 as ‘large’ and AUC > 0.8 as ‘very large’ [39, 41]. In addition, the difference in AUC between the different models was also estimated [39–41]. This was done using the DeLong method [43], with statistical significance being determined by the use of a 5% significance level. The differences in AUC were visualized by plotting the sensitivity against the false positive rate (1 – specificity) of the three models, allowing a visual comparison of the receiver operating characteristic (ROC) curves and AUC. Model fit was also examined using the Akaike Information Criterion (AIC) [40].

R version 4.3.1 was used for all statistical analyses and the production of the figures [44].

## Results

### Descriptive statistics

Of 8,823 children, 4,534 (51.4%) were boys and 4,289 (48.6%) were girls. The number of children who lived with one parent or had two parents born in another country was low, 661 (7.5%) and 307 (3.5%) respectively. The distribution of characteristics in the study population and the prevalence of social-emotional problems can be seen in Table 1.

**Table 1:** Distribution of characteristics and prevalence of social-emotional problems (ASQ:SE ≥ 59)

Characteristic	Overall *N* (Column %)	ASQ:SE$\:\ge\:$59 *N* (Row %)
Study participants	8,823 (100)	821 (9.3)
Child’s sex		
Girl	4,289 (48.6)	257 (6.0)
Boy	4,534 (51.4)	564 (12.4)
Custody arrangement		
Living with both parents	8,162 (92.5)	718 (8.8)
Not living with both parents	661 (7.5)	103 (15.6)
Place of Residence		
Södra Lappland	1,085 (12.3)	95 (8.8)
Skellefteå	2,334 (26.5)	220 (9.4)
Umeå	5,404 (61.2)	506 (9.4)
Parents’ income by tercile		
High	2,957 (33.5)	232 (7.8)
Middle	2,973 (33.7)	236 (7.9)
Low	2,893 (32.8)	353 (12.2)
Parents’ educational level		
Both more than high school	3,102 (35.2)	235 (7.6)
One more than high school	2,859 (32.4)	244 (8.5)
None more than high school	2,862 (32.4)	342 (11.9)
Parents’ place of birth		
Both born in Sweden	7,554 (85.6)	637 (8.4)
One born in Sweden	962 (10.9)	119 (12.4)
Both born outside Sweden	307 (3.5)	65 (21.2)

Among the children in the sample 821 (9.3%) had social-emotional problems. The prevalence was highest among those children whose parents were both born outside of Sweden at 21.2% (*n* = 65), followed by the custody situation of a child not living with both parents at 15.6% (*n* = 103) and being a boy at 12.4% (*n* = 564). Among children whose family income was in the lowest tercile and among children who had parents without more than a high school education, the prevalence of an ASQ:SE score of 59 or more was 12.2%) (*n* = 353) and 11.9% (*n* = 342) respectively. The prevalence of the outcome was similar in the three categories of place of residence.

**Fig. 2:**
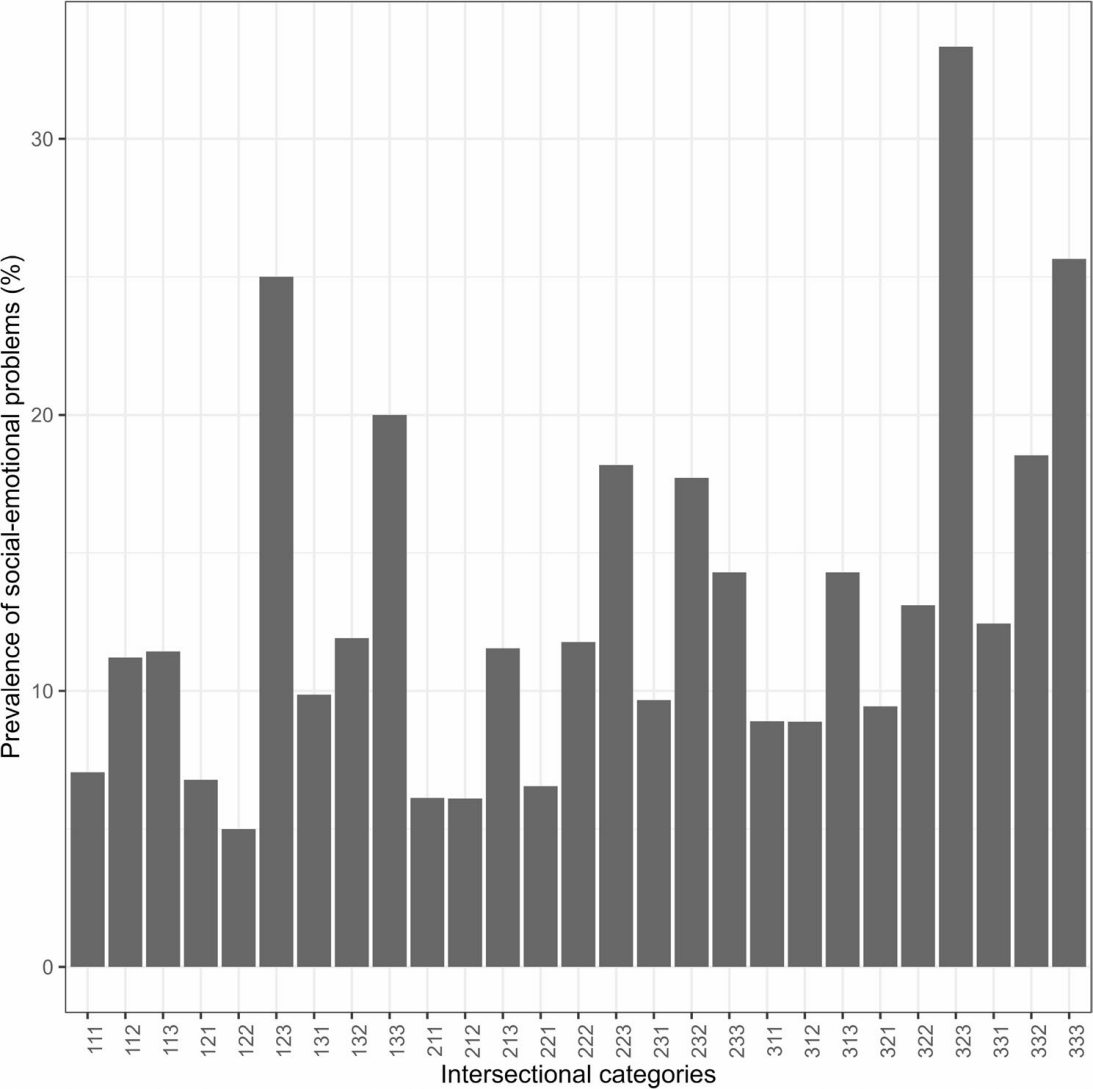
Prevalence of social-emotional problems (ASQ:SE ≥ 59) in the intersectional categories. In each intersectional category the first position in the number relates to parents’ income, the second position relates to parents’ education, and the third position relates to parents’ place of birth. The number 1 represents the most advantaged category and the number 3 the most disadvantaged category. As an example, category 123 includes children whose parents’ income was in the highest tercile, one parent had a higher educational level and whose parents were both born outside of Sweden

Regarding the intersectional cross-classified variable, the prevalence of a score of 59 or more was generally higher in the more disadvantaged groups. The prevalence of the outcome in the intersectional categories is visualized in Fig. 2 and ranged between 5.0% and 33.3%. Table 2 summarizes this information and also shows that the number of individuals was low in certain intersectional categories.

**Table 2:** Distribution of participants and social-emotional problems (ASQ:SE ≥ 59) in the intersectional categories

Characteristic	Overall *N* (Column %)	ASQ:SE ≥ 59 *N* (Row %)
Study Participants	8,823 (100)	821 (9.3)
Intersectional categories		
111	1,234 (14.0)	87 (7.1)
112	116 (1.3)	13 (11.2)
113	35 (0.4)	4 (11.4)
121	885 (10.0)	60 (6.8)
122	60 (0.7)	3 (5.0)
123	12 (0.1)	3 (25.0)
131	568 (6.4)	56 (9.9)
132	42 (0.5)	5 (11.9)
133	5 (0.1)	1 (20.0)
211	865 (9.8)	53 (6.1)
212	82 (0.9)	5 (6.1)
213	26 (0.3)	3 (11.5)
221	901 (10.2)	59 (6.5)
222	102 (1.2)	12 (11.8)
223	11 (0.1)	2 (18.2)
231	900 (10.2)	87 (9.7)
232	79 (0.9)	14 (17.7)
233	7 (0.1)	1 (14.3)
311	539 (6.1)	48 (8.9)
312	135 (1.5)	12 (8.9)
313	70 (0.8)	10 (14.3)
321	657 (7.4)	62 (9.4)
322	168 (1.9)	22 (13.1)
323	63 (0.7)	21 (33.3)
331	1,005 (11.4)	125 (12.4)
332	178 (2.0)	33 (18.5)
333	78 (0.9)	20 (25.6)

In each intersectional category the first position in the number relates to parents’ income, the second position relates to parents’ education, and the third position relates to parents’ place of birth. The number 1 represents the most advantaged category and the number 3 the most disadvantaged category. As an example, category 123 includes children whose parents’ income was in the highest tercile, one parent had a higher educational level and whose parents were both born outside of Sweden

### Estimates of social and intersectional inequalities

The three regression models estimating risk differences are displayed in Table 3 with 95% confidence intervals. In Model 1, an adjusted regression analysis which included only the covariates of child’s sex, place of residence and custody arrangement, significant risk differences were found of 6.4% (95% CI 5.2 to 7.6%) for boys compared to the reference category of girls and 6.6% (95% CI 3.8 to 9.4%) for children not living with both parents compared to living with both parents. Place of residence was not found to be significantly associated to the outcome in this model.

**Table 3:** Risk differences for social-emotional problems (ASQ:SE ≥ 59)

	Estimated Risk Difference % (95% CI)		
	Model 1	Model 2	Model 3
Child’s sex			
Girl	Reference	Reference	Reference
Boy	6.38 (5.20–7.56)	6.19 (5.05–7.32)	6.17 (5.04–7.30)
Custody arrangement			
Living with both parents	Reference	Reference	Reference
Not living with both parents	6.62 (3.84–9.39)	4.95 (2.15–7.75)	4.97 (2.17–7.77)
Place of residence			
Södra Lappland	Reference	Reference	Reference
Skellefteå	1.79 (-0.06-3.64)	2.25 (0.60–3.90)	2.19 (0.55–3.84)
Umeå	1.30 (-0.33-2.93)	2.10 (0.65–3.56)	2.10 (0.66–3.55)
Parents’ income by tercile			
High		Reference	
Middle		-0.92 (-2.12-0.28)	
Low		1.41 (-0.05-2.86)	
Parents’ education			
Both more than high school		Reference	
One more than high school		0.36 (-0.84-1.55)	
None more than high school		3.82 (2.38–5.26)	
Parents’ place of birth			
Both born in Sweden		Reference	
One born in Sweden		2.45 (0.45–4.45)	
Both born outside Sweden		11.33 (6.79–15.88)	
Intersectional categories			
111			Reference
112			3.38 (-2.09-8.85)
113			5.13 (-5.34-15.59)
121			0.51 (-1.50-2.53)
122			-1.30 (-6.12-3.53)
123			17.30 (-7.05-41.64)
131			3.04 (0.34–5.73)
132			5.11 (-4.29-14.51)
133			16.95 (-19.83-53.72)
211			-0.93 (-2.79-0.92)
212			-0.22 (-5.43-4.98)
213			4.27 (-7.68-16.22)
221			-0.83 (-2.66-1.01)
222			2.53 (-3.18-8.23)
223			9.70 (-11.86-31.27)
231			2.82 (0.56–5.08)
232			10.61 (2.24–18.97)
233			4.19 (-20.35-28.72)
311			1.83 (-0.78-4.45)
312			1.35 (-3.24-5.94)
313			6.39 (-1.63-14.41)
321			1.28 (-1.09-3.65)
322			3.66 (-1.11-8.44)
323			25.36 (13.71–37.01)
331			4.76 (2.37–7.14)
332			10.04 (4.26–15.82)
333			17.84 (8.09–27.58)

The three adjusted regression models with estimated risk differences for social-emotional problems (ASQ:SE ≥ 59) compared to reference categories and with 95% confidence intervals (95% CI). In each intersectional category the first position in the number relates to parents’ income, the second position relates to parents’ education and the third position relates to parents’ place of birth. The number 1 represents the most advantaged category and the number 3 the most disadvantaged category. As an example, category 123 includes children whose parents’ income was in the highest tercile, one parent had a higher educational level and whose parents were both born outside of Sweden

In Model 2, in which parents’ income by tercile, parents’ education and parents’ place of birth were included in addition to the covariates included in Model 1, parents’ income by tercile was not clearly related to the outcome. In the case of parents’ education and the category of none more than high school, a risk difference of 3.8% (95% CI 2.4 to 5.3%) was found, and the risk difference for having one parent or both parents born outside of Sweden was found to be 2.5% (95% CI 0.5 to 4.5%) and 11.3% (95% CI 6.8 to 15.9%) respectively.

**Fig. 3:**
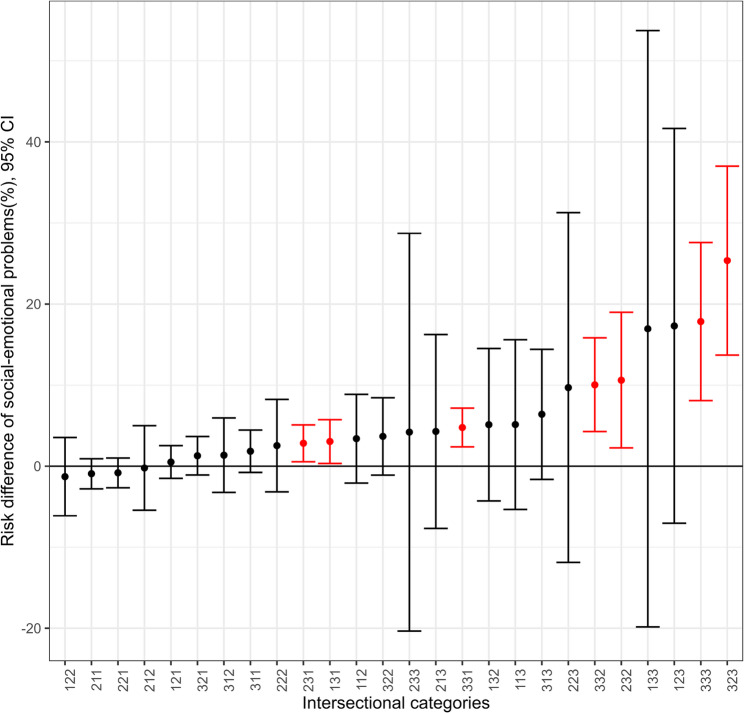
Risk differences of social-emotional problems (ASQ:SE ≥ 59) for the intersectional categories. Adjusted estimates of risk difference compared to the reference group of 111, in order of lowest to highest estimates. In each intersectional category the first position in the number relates to parents’ income, the second position relates to parents’ education, and the third position relates to parents’ place of birth. The number 1 represents the most advantaged category and the number 3 the most disadvantaged category. As an example, category 123 includes children whose parents’ income was in the highest tercile, one parent had a higher educational level and whose parents were both born outside of Sweden. 95% confidence intervals are provided and confidence intervals not including 0 are coloured red

Model 3 was an adjusted regression that included the same covariates as Model 1 and the intersectional cross-classified variable. Risk differences were estimated for the different intersectional categories compared to the reference category of 111, the category that included the most advantaged position for each of the three measured dimensions of social disadvantage: parents’ income, education and place of birth. In many of the intersectional categories the estimated risk differences were not found to be significant. Risk differences were generally higher for the more disadvantaged intersectional categories, i.e. children whose parents’ income was in the lower tercile, whose parents’ education was lower and where one or both parents were born outside of Sweden. For instance significant risk differences of 10.0% (95% CI 4.3 to 15.8) % were found for category 332, of 17.8% (95% CI 8.1 to 27.6%) for category 333 and as high as 25.4% (95% CI 13.7 to 37.0%) for category 323, children whose parents’ income was in the lowest tercile, one parent had a higher educational level and whose parents were both born outside of Sweden. Estimates of adjusted risk differences with 95% confidence intervals for the different intersectional categories are visualized in Fig. 3.

### Discriminatory accuracy

**Fig. 4:**
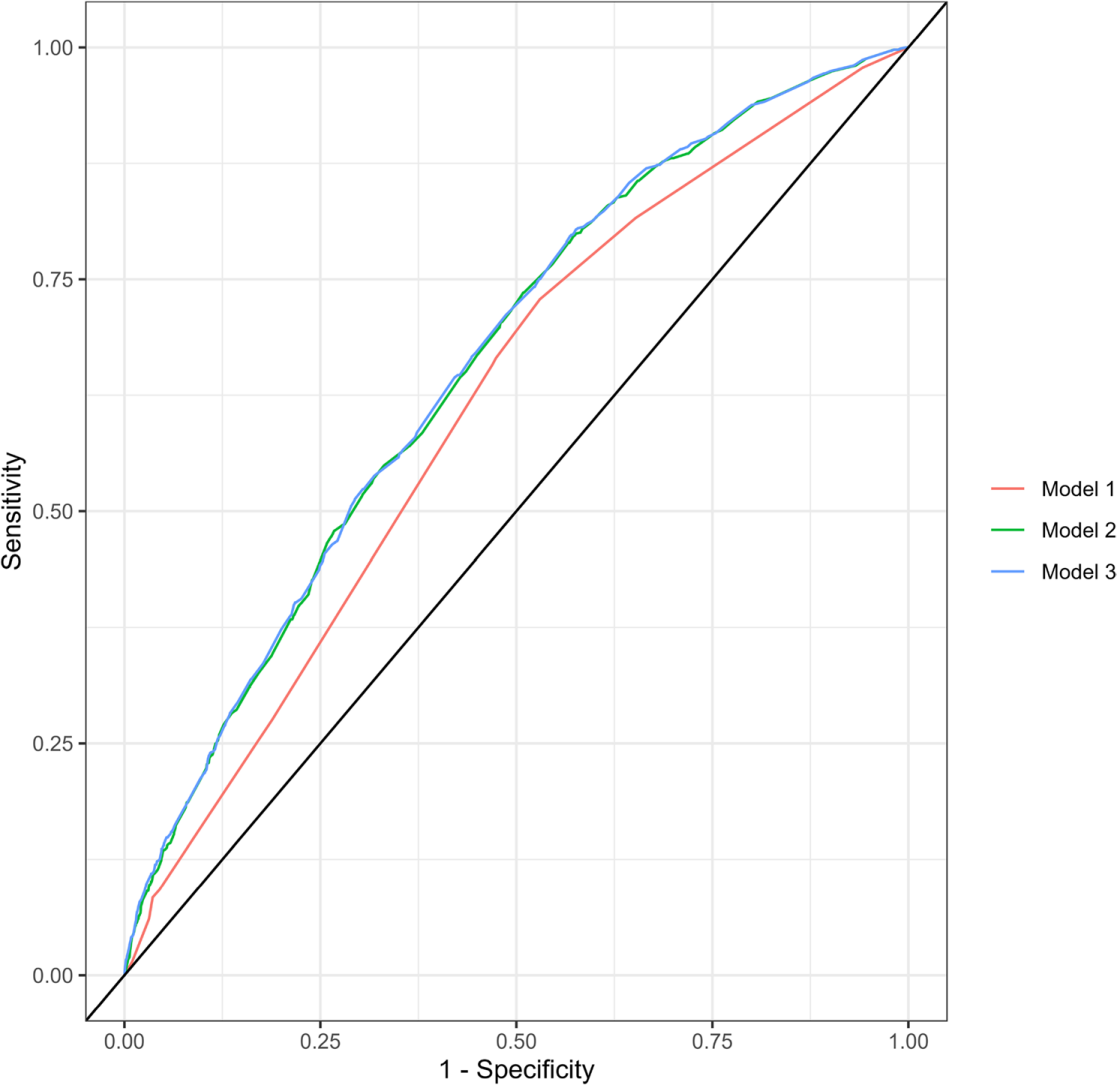
Receiver operating characteristic (ROC) curves for the three regression models. Model 1 includes only the covariates child’s sex, custody arrangement and place of residence. Model 2 consists of the single dimensions of social disadvantage in addition to the covariates. Model 3 consists of the intersectional variable in addition to the covariates

AUC for Model 1, which included only the covariates of child’s sex, custody arrangement and place of residence, was 0.613 indicating a moderate discriminatory accuracy. AUC improved in a statistically significant way with both the addition of the single dimensions of social disadvantage: parents’ income, education and place of birth (Model 2, AUC = 0.656), and with the addition of the intersectional variable (Model 3, AUC = 0.659) compared to Model 1. The discriminatory accuracy for both Model 2 and Model 3 was still interpreted as moderate. AUC and thereby discriminatory accuracy also improved when comparing Model 3 with Model 2 by 0.002 (95% CI -0.002 to 0.007), although the difference was not statistically significant. These results can be found in Table 4. In Fig. 4 the sensitivity and the false positive rate (1 – specificity) of the three models have been plotted against each other, demonstrating the differences between the receiver operating characteristic (ROC) curves, and thereby the AUC, of all models (Table 4).

**Table 4:** Discriminatory accuracy of the three regression models

	AUC	ΔAUC (95% CI)
Model 1^a^	0.613	
Model 2^b^	0.656	
Model 3^c^	0.659	
Model 1^a^ vs. Model 2^b^		0.043 (0.028–0.058)
Model 1^a^ vs. Model 3^c^		0.045 (0.031–0.060)
Model 2^a^ vs. Model 3^b^		0.002 ( -0.002–0.007)

Discriminatory accuracy measured as Area Under the Receiver Operating Characteristic Curve (AUC) and the difference in AUC(ΔAUC) between the models with 95% confidence intervals (95% CI)

^a^Model 1: Covariates: gender; custody arrangement; place of residence

^b^Model 2: Covariates in Model 1 + parents’ income; parents’ education; parents’ place of birth

^c^Model 3: Covariates in Model 1 + the intersectional variable

## Discussion

The aim of this study was to examine intersectional inequalities in social-emotional problems in three-year-old children in addition to considering the public health implications. Our main findings are that average risk differences of social-emotional problems in three-year-olds tended to increase in intersectional groups where multiple dimensions of social disadvantage overlapped, especially in relation to parents’ place of birth. The discriminatory accuracy of all three included models was estimated as moderate, indicating a need for a public health focus on the whole population rather than solely focusing on those groups with the dimensions of social disadvantage included in this study. It did however improve in a slight but statistically significant way with the addition of dimensions of social disadvantage compared to the model with only the specified covariates, implying that including such dimensions of social disadvantage can contribute to discriminatory ability when it comes to preschool children’s social-emotional problems in this context. Finally, the AUC of the model including the intersectional approach did not differ significantly from the model including unidimensional social inequalities, which could suggest that such an approach does not necessarily discriminate better between social-emotional problems in preschool children compared to the more conventional approach to social inequality.

This study contributes with an intersectional perspective to mental health in early childhood, a field where there is still a general lack of knowledge. A relatively recent meta-analysis of children aged 1–7 years [45], concluded that there is a need for more research on the mental health of young children and its epidemiology, and a newly published report from the Public Health Agency of Sweden discusses a lack of national monitoring and a need to increase what is known with regards to the mental health of children aged 0–5 in Sweden [46]. Examining social-emotional problems in young children from an intersectional perspective increases the detail and nuance in understanding of how the distribution of this issue is related to the included social determinants of health and their intersections, compared to our previous findings [18]. Although research with an intersectional perspective is limited when it comes to mental health in early childhood, such research later in childhood and concerning adults also seems to provide opportunities to increase understanding of social and intersectional inequalities [47–50]. In relation to the discriminatory accuracy of such approaches to mental health, findings have also pointed to relatively low discriminatory accuracy of intersectional groups [47] and universal public health initiatives being more effective than those focusing on more specific groups with higher risk [48].

The findings in this study are in line with previous research on the association between socioeconomic factors such as family income or parental educational level and social-emotional and behavioural problems in young children in international contexts [14–17, 19, 20] and in preschool children in Swedish contexts [21, 24]. With regards to parental immigrant background, results do not seem to be consistent across those studies that have included it, with some findings contrasting the results of this study. It has been found to be associated with mental health and social-emotional problems in young children in Sweden [21] and the Netherlands [20] and English as a second language has been similarly linked to social-emotional competence in Canada [14], but mental health has also been found to be comparable between children with parents born in Sweden and parents born outside of Europe [24]. In another Canadian context immigrant background has been found to be associated with lower odds for indicators related negatively to emotional development and lower odds for medical appointments for mental health problems in childhood [51] and in the United States kindergarten children of immigrant families were found to have higher social-emotional competence [52]. In a systematic review of Scandinavian countries, ethnic inequalities in mental health outcomes in children and adolescents were found to be largely explained by socioeconomic status in several studies [53], while the results of this study indicate a greater magnitude of risk for social-emotional problems when socioeconomic disadvantage and immigrant background intersect. Children with an immigrant background are a group within which it is likely that there is large heterogeneity, especially when comparing different contexts. It is also important to keep in mind the limitations involved when examining the association between parents’ country of birth and social-emotional problems, as there may be both cultural context as well as language barriers influencing findings. Mock-Muñoz de Luna et al. also suggest that health inequalities with regards to ethnicity and immigrant background could for instance be related to factors such as discrimination or the migration experience [53].

### Methodological considerations

The sample used in this study is population-based with a high response rate of about 71% of all three-year-old children living in the county, reducing the risk of selection bias. The use of high-quality individual-level register data for the included dimensions of social disadvantage rather than self-reported data further increases the validity of findings. This is an observational study with a cross-sectional design which inherently restricts the conclusions that can be drawn, and the findings should not be interpreted as causal.

The ASQ:SE has been evaluated in this specific context in Västerbotten County [34, 35], but there is still a risk of response bias since it is a questionnaire completed by the parent or caregiver. Cultural context and language barriers may have affected accurate data collection for parents born outside Sweden, in relation to how ASQ:SE items are interpreted, and the possibility that there are cultural differences in how children’s behaviour is viewed. Despite the strengths in this study’s methods of sampling and data collection considering most Swedish children are covered by Child Health Services [54], cultural context and language barriers may also have contributed to families with an immigrant background being underrepresented in our sample. In particular as an effect of the ASQ:SE only being available in Swedish and English. The proportion of children with two foreign-born parents in our study also does appears to be lower when compared with regional population statistics for the same period of time and the proportion of children with lower educated parents slightly lower when compared with national population statistics. The information on the sociodemographic characteristics of those children and their families who did not participate in this study or were excluded is limited, although there is evidence from another Swedish county that disadvantaged families receive basic child health services equally to more advantaged families [55]. There may however still be an overrepresentation of disadvantaged sociodemographic characteristics, such as immigrant background, among those children whose parents did not respond to the Salut questionnaire, did not give consent for the study, where there was no personal identification number or where information was missing from national population registers and the Salut questionnaire. With these limitations in mind, this population-level monitoring and data collection should have produced a sample that is sufficiently representative of Västerbotten County. These findings may also be generalisable to other counties in Sweden and countries in Scandinavia, considering the relative homogeneity with regards to demography, social patterning and health care accessibility, although the same limitations should be kept in mind.

Pooled analysis on data from the years of 2014–2108 was conducted and no information was available on whether included participants were siblings, resulting in the possibility that some observations included in the analysis are not independent. It is considered unlikely though that this would have a substantial effect on results. Participants with missing data in relevant variables were also excluded, but the effect of this was determined to be negligible as the distribution demonstrated a sufficiently random pattern.

The use of an additive model and consideration of additive-scale interactions rather than multiplicative-scale interactions, has been argued to be more consistent with intersectionality [25] as well as being more relevant when it comes to informing public health policy [56]. Another methodological strength of this study is the use of an AIHDA approach which shares important similarities with its’ multilevel counterpart MAIHDA with regards to quantitative research with an intersectional approach [39]. MAIHDA has been argued to be methodologically as well as theoretically well suited to quantitative intersectional research as it statistically take the clustering that is associated with social groups into account as well as viewing individuals as situated within intersectional categories rather than focusing on individual-level factors [57]. Advantages of using AIHDA are that it is perceived as more understandable than MAIHDA, while still comprehensively charting health inequalities compared to conventional unidimensional approaches and potentially increasing the public health relevance of findings by including discriminatory accuracy [39]. By focusing less on average estimates of risk, unwarranted stigmatization of population groups may also be avoided [30].

The choice of an intersectional approach in this study resulted in a small number of participants in certain intersectional groups. This affects the extent to which conclusions can be drawn regarding these groups. If the number of participants with certain sociodemographic characteristics had been greater in for instance a larger sample, this could have improved the study. It is also possible that the use of multilevel analysis such as MAIHDA could have produced more reliable estimates, although the chosen approach AIHDA has the previously mentioned advantage. If further categorisation of the included dimensions of social disadvantage or the addition of other social determinants of health had been supported by sample size, this could also have improved the intersectional categorisation and mapping, possibly increasing the discriminatory accuracy of the model including intersectional categories [58]. This might for instance include a more precise categorisation of parents’ place of birth or the inclusion of migration reason or experience for those parents born outside of Sweden. It is worth noting that parents’ income, when categorised by tercile, was not found to be significantly related to the outcome although a categorisation by quintile was found to be significantly related to the outcome in the lowest income quintile in our previous work [18].

### Public health implications and future research

Proportionate universalism is concerned with addressing health inequalities by not only targeting the most vulnerable populations, but instead through combining universal and targeted policies in order to ensure that the public health action taken is proportionate to need across the social gradient that exists within health [59]. There may however be challenges associated with practically applying proportionate universalism, for instance when it comes to policy design with regards to achieving proportionality [60]. Intersectional approaches to health inequalities that include the measurement of discriminatory accuracy, such as an analysis of individual heterogeneity and discriminatory accuracy (AIHDA) approach, have been put forward as a way of addressing this issue by offering a method of determining to what degree universal programs should be combined with targeted public health interventions for specific health problems [39, 41].

The findings of this study suggest that public health interventions directed at preventing and decreasing social-emotional problems in young children should be universal but that there is also a need for some extent of proportionate action with regards to the social determinants of health examined. Although the academic literature is limited on intersectional inequalities in social-emotional problems in early childhood and the discriminatory accuracy of such approaches, based on this study there seems to be a need for universal child health promotion programs such as the Salut Child Health Intervention Program. Additionally, some degree of targeted action when it comes to vulnerable groups seems warranted, also taking into consideration the magnitude of risk difference found for instance in relation to parents’ place of birth, although this needs to be further evaluated.

The discriminatory accuracy of models including social and intersectional inequalities with regards to social-emotional problems was in this study moderate, although there was a significant difference compared to the model including only the specified covariates. Classification of discriminatory accuracy in relation to determining public health policy is still lacking and should therefore be interpreted with caution so that intersectional and social health inequalities are not arbitrarily disregarded, as highlighted by Gustafsson et al. [40]. Mulinari et al. also emphasize that information other than discriminatory accuracy must be taken into account as it may be relevant when considering public health interventions [61].

There is a need for further understanding of inequalities in social-emotional problems in young children, particularly in relation to children with an immigrant background. Additional validation of the ASQ:SE with regards to cultural context through for instance qualitative studies and the use of reliably translated versions of the instrument could help overcome bias related to cultural context or language barriers. Qualitative studies could also shed light on the influence of cultural context and the higher prevalence of social-emotional problems among children with an immigrant background. Including important confounders such as family history of mental health problems [22] as well as a more detailed categorisation of the included dimensions of social disadvantage, especially with regards to parents’ country of birth, could also provide valuable information helping guide public health policy. Depending on future findings, it may also be warranted to examine the pathways by which social determinants of health such as immigrant background or the intersections of social inequalities are associated with social-emotional problems in young children.

## Conclusions

This study increases our understanding of intersectional and complex social inequalities in social-emotional problems in three-year-old children in Sweden. Although the number of participants in some intersectional categories was small limiting the conclusions that can be drawn, risk differences of the outcome were significant in groups where multiple dimensions of social disadvantage intersected, in particular relating to parents’ country of birth.

The results of this study can be applied in a way that is consistent with the concept of proportionate universalism and support the need for universal public health policies when it comes to social-emotional problems in young children in addition to policies targeting more vulnerable groups. An intersectional perspective and research that takes individual heterogeneity and discriminatory accuracy into consideration rather than merely estimating group averages can be important tools for increasing our knowledge of health inequities and more effectively approaching the improvement of population health.

## Supplementary Information

Supplementary Material 1.

## Data Availability

The datasets analysed in this study are not publicly available. They were originally collected by Region Västerbotten for a child health survey (https://www.regionvasterbotten.se/salut) and approval from both Region Västerbotten and the Ethical Vetting board was required before access to the data was granted for the current study. Requests to access the data for replication analyses are possible and should be directed to https://www.regionvasterbotten.se/salut.

## Abbreviations

95% CI: 95% Confidence interval
AIC: Akaike Information Criterion
AIHDA: Analysis of individual heterogeneity and discriminatory accuracy
ASQ: SE: Ages and Stages Questionnaires: Social-Emotional
AUC: Area under the receiver operating characteristics curve
MAIHDA: Multilevel analysis of individual heterogeneity and discriminatory accuracy
ROC: Receiver operating characteristics
