# Correction: Intersectional inequalities in social-emotional problems among three-year-old children in Sweden: a population-based study

**DOI:** 10.1186/s12889-026-27740-3

**Published:** 2026-05-16

**Authors:** Ana Rosenberg, Anneli Ivarsson, Marie Lindkvist, Sven-Arne Silfverdal, Masoud Vaezghasemi

**Affiliations:** 1 Department of Epidemiology and Global Health, Umeå University, Umeå, Sweden; 2 Department of Clinical Science, Pediatrics, Umeå University, Umeå, Sweden

**Correction: BMC Public Health 26, 1236 (2026)**

**https://doi.org/10.1186/s12889-026-27220-8**

Following publication of the original article [1], the authors reported an error in the citation of Figs. 2, 3 and 4 within the Statistical analysis section and in Table 4. The citations of Figs. 2, 3 and 4 have been removed.

In Table 4, the rows “Model 1a vs. Model 2b; Model 1a vs. Model 3c; and Model 2a vs. Model 3b”, the data currently under “AUC” should be moved to “ΔAUC (95% CI)”. The correct Table 4 is presented below:

**Table 4:** Discriminatory accuracy of the three regression models

Model 1^a^	AUC	ΔAUC (95% CI)
	0.613	
Model 2^b^	0.656	
Model 3^c^	0.659	
Model 1^a^ vs. Model 2^b^		0.043 (0.028–0.058)
Model 1^a^ vs. Model 3^c^		0.045 (0.031–0.060)
Model 2^a^ vs. Model 3^b^		0.002 (-0.002–0.007)

Discriminatory accuracy measured as Area Under the Receiver Operating Characteristic Curve (AUC) and the difference in AUC(ΔAUC) between the models with 95% confidence intervals (95% CI)

^a^Model 1: Covariates: gender; custody arrangement; place of residence

^b^Model 2: Covariates in Model 1 + parents’ income; parents’ education; parents’ place of birth

^c^Model 3: Covariates in Model 1 + the intersectional variable

The original article [1] has been updated.

## References

[1] Rosenberg A, Ivarsson A, Lindkvist M, et al. Intersectional inequalities in social-emotional problems among three-year-old children in Sweden: a population-based study. BMC Public Health. 2026;26:1236. 10.1186/s12889-026-27220-8. 41957602 10.1186/s12889-026-27220-8 PMC13085497

