# Supplementary material for: Intersectional inequalities in social-emotional problems among three-year-old children in Sweden: a population-based study
Source: BMC Public Health. 2026 Apr 9;26:1236. doi: 10.1186/s12889-026-27220-8 (PMC13085497; doi:10.1186/s12889-026-27220-8)
Supplement: Supplementary file 1 — Supplementary Material 1. [file 12889_2026_27220_MOESM1_ESM.docx]

Additional File 1

Results Using the ASQ:SE Cut-off of 50

**Supplementary table 1. Risk differences for social-emotional problems (ASQ:SE** $\boldsymbol{\geq}$**50).**

|  | Estimated Risk Difference % (95% CI) | | | | | | |
| --- | --- | --- | --- | --- | --- | --- | --- |
|  | Model 1 | | | Model 2 | | Model 3 | |
| Child’s sex |  |  | |  | |  |  |
| Girl | Reference | | | Reference | | Reference | |
| Boy | 9.00 | (7.51-10.49) | | 8.83 | (7.38-10.28) | 8.83 | (7.38-10.28) |
| Custody arrangement |  |  | |  |  |  |  |
| Living with both parents | Reference | | | Reference | | Reference | |
| Not living with both parents | 7.45 | (4.22-10.69) | | 5.08 | (1.8-8.36) | 5.16 | (1.87-8.44) |
| Place of residence |  |  | |  |  |  |  |
| Norra Lappland | Reference | | | Reference | | Reference | |
| Skellefteå | 3.21 | (0.82-5.60) | | 4.38 | (2.14-6.62) | 4.14 | (1.89-6.39) |
| Umeå | 2.59 | (0.48-4.71) | | 4.34 | (2.34-6.35) | 4.17 | (2.16-6.19) |
| Parents’ income by tercile |  |  | |  |  |  |  |
| High |  |  | | Reference | |  |  |
| Middle |  |  | | -0.75 | (-2.39-0.89) |  |  |
| Low |  |  | | 2.12 | (0.24-4.00) |  |  |
| Parents’ education |  |  | |  |  |  |  |
| Both more than high school |  |  | | Reference | |  |  |
| One more than high school |  |  | | 5.59 | (2.97-8.22) |  |  |
| None more than high school | | |  | 16.69 | (11.42-21.97) |  |  |
| Parents’ place of birth |  |  | |  |  |  |  |
| Both born in Sweden |  |  | | Reference | |  |  |
| One born in Sweden |  |  | | 1.04 | (-0.61-2.69) |  |  |
| Both born outside Sweden |  |  | | 5.11 | (3.26-6.96) |  |  |
| Intersectional categories |  |  | |  |  |  |  |
| 111 |  |  | |  |  | Reference | |
| 112 |  |  | |  |  | 8.53 | (1.07-16.00) |
| 113 |  |  | |  |  | 6.62 | (-6.04-19.28) |
| 121 |  |  | |  |  | 0.79 | (-1.90-3.48) |
| 122 |  |  | |  |  | 5.91 | (-3.65-15.46) |
| 123 |  |  | |  |  | 28.54 | (0.71-56.38) |
| 131 |  |  | |  |  | 3.52 | (0.15-6.89) |
| 132 |  |  | |  |  | 5.73 | (-5.42-16.88) |
| 133 |  |  | |  |  | 31.36 | (-11.64-74.36) |
| 211 |  |  | |  |  | -1.41 | (-3.96-1.15) |
| 212 |  |  | |  |  | 0.74 | (-6.55-8.03) |
| 213 |  |  | |  |  | 8.79 | (-6.79-24.38) |
| 221 |  |  | |  |  | 0.32 | (-2.33-2.98) |
| 222 |  |  | |  |  | 7.21 | (-0.62-15.05) |
| 223 |  |  | |  |  | 4.20 | (-16.9-25.3) |
| 231 |  |  | |  |  | 4.24 | (1.32-7.16) |
| 232 |  |  | |  |  | 13.39 | (3.75-23.03) |
| 233 |  |  | |  |  | 11.94 | (-20.77-44.65) |
| 311 |  |  | |  |  | 2.48 | (-0.89-5.85) |
| 312 |  |  | |  |  | 7.42 | (0.57-14.28) |
| 313 |  |  | |  |  | 12.36 | (2.16-22.56) |
| 321 |  |  | |  |  | 1.58 | (-1.49-4.65) |
| 322 |  |  | |  |  | 6.26 | (0.19-12.32) |
| 323 |  |  | |  |  | 33.11 | (20.72-45.50) |
| 331 |  |  | |  |  | 7.16 | (4.13-10.18) |
| 332 |  |  | |  |  | 13.14 | (6.45-19.84) |
| 333 |  |  | |  |  | 25.38 | (14.48-36.28) |

The three adjusted regression models with estimated risk differences for social-emotional problems (ASQ:SE $\boldsymbol{\geq}$50) compared to reference categories and 95% confidence intervals (95% CI). In each intersectional category the first position in the number relates to parents’ income, the second position relates to parents’ education and the third position relates to parents’ place of birth. The number 1 represents the most advantaged category and the number 3 the most disadvantaged category. As an example, category 123 includes children whose parents’ income was in the highest tercile, one parent had a higher educational level and whose parents were both born outside of Sweden.

**Supplementary table 2. Discriminatory accuracy of the three regression models**

|  | AUC | ΔAUC (95% CI) | |
| --- | --- | --- | --- |
| Model 1^a^ | 0.602 |  |  |
| Model 2^b^ | 0.637 |  |  |
| Model 3^c^ | 0.639 |  |  |
| Model 1^a^ vs Model 2^b^ |  | 0.036 | (0.024-0.047) |
| Model 1^a^ vs Model 3^c^ |  | 0.038 | (0.026-0.050) |
| Model 2^a^ vs Model 3^b^ |  | 0.002 | (-0.001-0.006) |
| ^a^ Model 1: Covariates: child’s sex; custody arrangement; place of residence  ^b^ Model 2: Covariates in Model 1 + parents’ income; parents’ education; parents’ place of birth  ^c^ Model 3: Covariates in Model 1 + the intersectional variable | | | |

Discriminatory accuracy of the three models, with the outcome defined as ASQ:SE $\boldsymbol{\geq}$50, measured as Area Under the Receiver Operating Characteristic Curve (AUC) and the difference in AUC between the models with 95% confidence intervals (95% CI).
